# hTERT mediates gastric cancer metastasis partially through the indirect targeting of ITGB1 by microRNA-29a

**DOI:** 10.1038/srep21955

**Published:** 2016-02-23

**Authors:** Bing He, Yu-Feng Xiao, Bo Tang, Yu-Yun Wu, Chang-Jiang Hu, Rui Xie, Xin Yang, Song-Tao Yu, Hui Dong, Xiao-Yan Zhao, Ji-Liang Li, Shi-Ming Yang

**Affiliations:** 1 Department of Gastroenterology, Xinqiao Hospital, Third Military Medical University, Chongqing, 400037, P.R. China; 2 Department of Oncology and Southwest Cancer Center, Southwest Hospital Third Military Medical University, Chongqing, 400037, P.R. China; 3 Institute of Translational and Stratified Medicine, Plymouth University Peninsula Schools of Medicine and Dentistry, The John Bull Building, 16 Research way, Plymouth PL68BU, UK

## Abstract

Human telomerase reverse transcriptase (hTERT) plays a key role in tumor invasion and metastasis, but the mechanism of its involvement in these processes is not clear. The purpose of this study is to investigate the possible molecular mechanism of hTERT in the promotion of gastric cancer (GC) metastasis. We found that the up-regulation of hTERT in gastric cancer cells could inhibit the expression of miR-29a and enhance the expression of Integrin β1 (ITGB1). In addition, the invasive capacity of gastric cancer cells was also highly increased after hTERT overexpression. Our study also found that the restoration of miR-29a suppressed the expression of ITGB1 and inhibited GC cell metastasis both *in vitro* and *in vivo*. Taken together, our results suggested that hTERT may promote GC metastasis through the hTERT-miR-29a-ITGB1 regulatory pathway.

Gastric cancer (GC) is one of the most common malignant diseases and is responsible for a considerable portion of cancer-related mortality^1^. Despite the studies that have been conducted in previous years, the molecular mechanism of gastric cancer has yet to be elucidated. Previous studies have shown that cell externalization is an initial sign of malignant transformation^2^. Telomerase, which is responsible for telomere lengthening, is a key factor in the processes of cell externalization and malignant transformation^3^,^4^. It is activated in approximately 90% of cancer cells, including gastric cancer cells^5^. hTERT is the limiting subunit of telomerase and plays a decisive role in the activation of telomerase. The expression level of hTERT has been positively correlated with telomerase activity^6^. Many studies have shown that hTERT is related to the unlimited growth of gastric cancer cells^7^,^8^. In addition, our previous study found that hTERT was highly expressed in GC tissues, we also proved that hTERT was positive related to the distant metastasis and lymphatic metastasis in patients with GC^9^. The overexpression of hTERT can significantly enhance the invasiveness and metastatic ability of the telomerase-negative osteosarcoma cell line U2OS^10^. Other studies have indicated that hTERT can increase the invasive ability of cancer cells^11^,^12^. However, the mechanism by which hTERT regulates the invasion and metastasis of gastric cancer is unclear.

ITGB1 (Integrin β1) is a type of cell adhesion molecule whose main function is to mediate mutual adhesion between cells and the ECM (extracellular matrix). ITGB1 is often expressed abnormally in cancer and correlates with malignant tumor phenotypes, such as invasion, migration, angiogenesis and proliferation^13^. Recent studies have also reported that ITGB1 can enhance the secretion and activation of matrix metalloproteinases 14 (MMP14)^14^, decrease the adhesiveness between tumor cells, promote the detachment of tumor cells from the tumor body, and enhance adhesion between tumor cells and the ECM^15^. Interestingly, in the present study, using proteomic technology, we found that hTERT can increase ITGB1 expression when exogenous hTERT is overexpressed in U2OS cells. However, hTERT is not a nuclear transcription factor and therefore cannot directly regulate ITGB1. Our previous study found that ITGB1 could be regulated by hTERT by enhancing FOXO3a ubiquitination^16^, we then hypothesis that mircroRNAs may also play an important role in the regulation of ITGB1.

MicroRNAs play key roles in tumor metastasis^17^. A number of studies have shown that microRNAs can regulate a number of tumor-related transcription factors that are involved in tumor invasion and proliferation^18^. Studies have shown that some miRNAs can regulate the expression of ITGB1^19^,^20^, and thereby reduce the ability of tumor cells to invade and metastasize. Thus, we hypothesized whether hTERT could regulate ITGB1 through miRNA(s).

In the present study, we reported that hTERT can decrease miRNA-29a levels to indirectly enhance ITGB1 expression, which triggers tumor metastasis. We hope that this study can supplement previous data in describing how hTERT might enhance the malignant activity of gastric cancer cells.

## Results

### hTERT regulates ITGB1 in GC

Because U2OS cells do not express endogenous hTERT, we used these cells to analyze the changes in protein after the overexpression of hTERT. We used a proteomics approach to analyze the protein expression in U2OS-hTERT and U2OS-Vector cells, and then a Gene Set Enrichment Analysis was used to analyze the proteomic results. Our previous study has shown that the fold change of ITGB1 was the highest^16^ (Supplementary Fig. 1).

**Figure 1:**
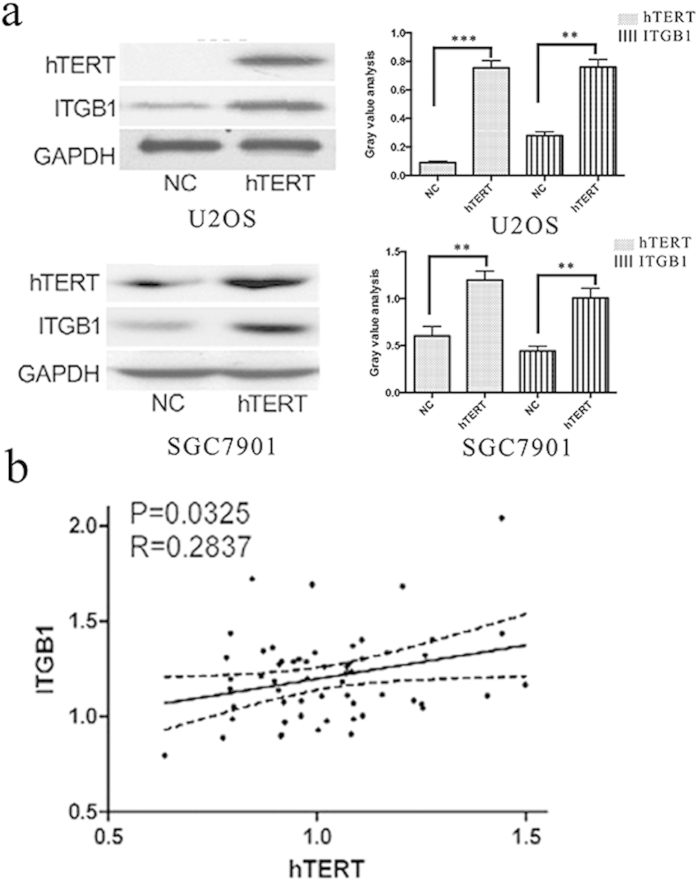
ITGB1 is regulated by hTERT in GC. (**a**) The expression of ITGB1 was assessed by WB after hTERT was overexpressed in U2OS cells and in the human GC cell line SGC7901. ***P < 0.001; **P < 0.01. (**b**) The correlation between the protein levels of hTERT and ITGB1 as determined by immunostaining of gastric cancer and normal tissues. Image-Pro Plus 6.0 was used to analysis the relative radio of immunostaining.

We then used western blot to validate the proteomic results. By western blot, we found that ITGB1 expression was significantly increased in U2OS-hTERT and gastric cancer cell line SGC7901, in which hTERT was overexpressed (Fig. 1a). In addition, in clinical GC specimens the expression level of hTERT was positively correlated with ITGB1 expression (Fig. 1b), which indicates that ITGB1 may be regulated by hTERT in GC.

### hTERT may regulate ITGB1 expression via miR-29, which binds to the 3′UTR of ITGB1

Our previous study has shown that hTERT cannot promote ITGB1 expression directly^16^. Recent studies have reported that ITGB1 can be regulated by miRNAs, which would reduce the invasiveness and metastatic ability of cancer cells^21^,^22^. Therefore, we hypothesized whether ITGB1 expression is regulated by hTERT via the control of miRNA(s), which would trigger tumor metastasis.

**Figure 2:**
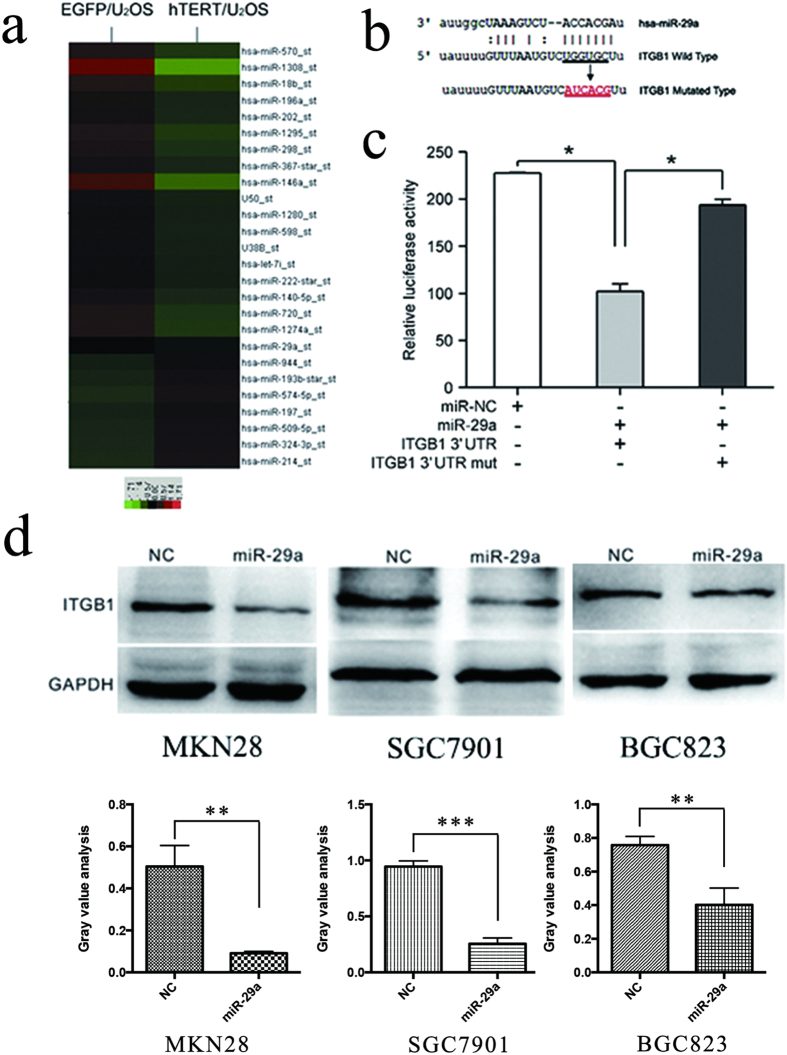
hTERT may regulate ITGB1 expression via miR-29, which binds to the 3′UTR of ITGB1. (**a**) A microRNA array was used to detect the differentially expressed miRNA in hTERT/U2OS and EGFP/U2OS cells. (**b**) The predicted miR-29a binding seed sequence in ITGB1, and the sequence was mutated by site directed mutagenesis. (**c**) The relative activities of the Firefly and Renilla luciferase genes were assayed in HEK293 cells 24 h after co-transfection with different miRNAs and the ITGB1 3′UTR or the mutated ITGB1 3′UTR (n = 3, unpaired t test). (**d**) The expression level of ITGB1 was examined after GC cells were transfected with miR-29a or control mimics.

Based on this hypothesis, miRNA microarray tests were used to scan the hTERT-regulating miRNA. A total of 153 miRNAs were found that may be regulated by hTERT (Fig. 2a and Supplementary Table 1). We also used bioinformatics technology to predict which miRNAs might regulate the expression of ITGB1. Bioinformatics analyses revealed that miR-29, miR-124, miR-183 and miR-506 might regulate the expression of ITGB1. Interestingly, miR-29 was the only miRNA that was found to regulate ITGB1 expression by bioinformatics analyses. Its expression was down-regulated in U2OS-hTERT cells (Fig. 2a). These results indicated that miR-29 may play a causal role in the process of hTERT regulation of ITGB1expression and the process of GC metastasis.

As mentioned above, our results indicated that hTERT may regulate ITGB1 expression via miR-29, but this hypothesis needed confirmation by further experiments.

The miR-29 family is composed of three family members (miR-29a, miR-29b and miR-29c), and they all share the same seed region. To determine whether miR-29 modulates ITGB1 directly, we cloned the 3′UTR of ITGB1 into luciferase reporters and co-transfected either miR-29 (miR-29a, miR-29b and miR-29c) or a control mimic. These assays showed that miR-29 family members (miR-29a, miR-29b and miR-29c) significantly decreased the luciferase activity (Fig. 2c, Supplementary Fig. 2a and Supplementary Fig. 2b). We also generated a mutant reporter (Luc-ITGB1-mu), in which the predicted miR-29 binding site to ITGB1 was mutated (ITGB1-mu) (Fig. 2b). Then, it was revealed that the luciferase activity was partially relieved after transfection with miR-29 mimics (Fig. 2c), which suggests that miR-29 decreased the luciferase activity of Luc-ITGB1 but did not affect Luc-ITGB1-mu. We also found by western blot that the protein levels of ITGB1 decreased after the introduction of miR-29 family members mimics, which indicates that miR-29 reduced the protein expression level of ITGB1 (Fig. 2d and Supplementary Fig. 3).

Considering these results, we concluded that miR-29 reduced the expression of ITGB1 via the direct targeting of the 3′UTR of ITGB1.

### miR-29a and ITGB1 are negatively correlated in clinical GC specimens

**Figure 3:**
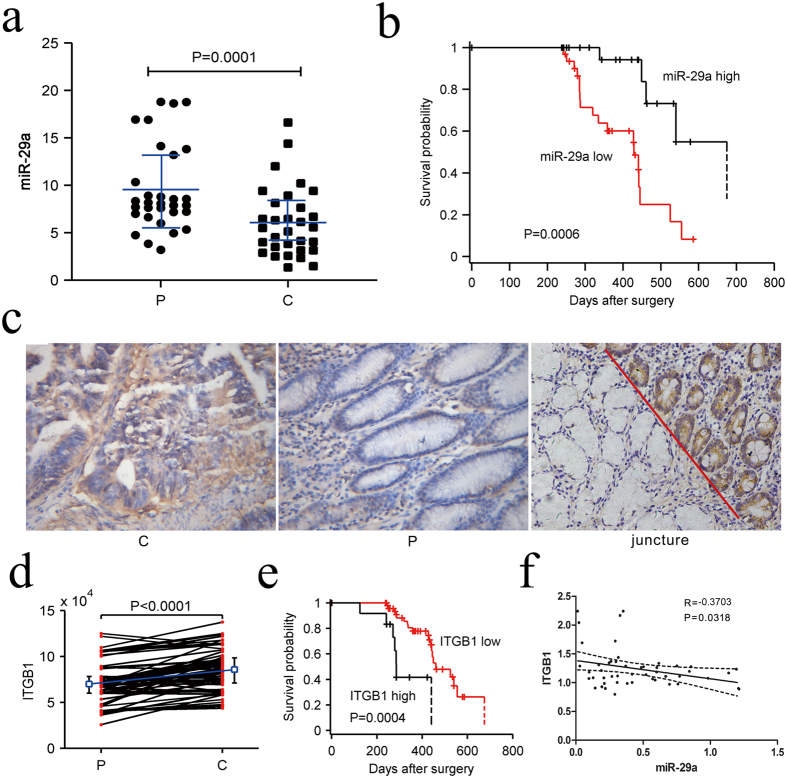
miR-29a and ITGB1 are inversely correlated in clinical GC specimens. (**a**) The expression levels of miR-29a in GC tissues (C) and paracancerous tissues adjacent to the tumor (P) by qRT-PCR. (**b**) Kaplan–Meier survival curve and log-rank test for GC patients with high and low miR-29a expression. (**c,d**) The expression levels of ITGB1 were tested by IHC (C: GC tissues, P: paracancerous tissues adjacent to the tumor, juncture: the boundary between C and P). (**e**) Kaplan–Meier survival curve and log-rank tests for GC patients with high and low ITGB1 expression. (**f**) The correlation between the levels of miR-29a and ITGB1 in gastric cancer and in paracancerous tissues adjacent to the tumor.

The above results indicated that miR-29 family members may play a critical role in the regulation of ITGB1 expression by hTERT. We then examined the expression of miR-29 and ITGB1 in the tumors of patients with gastric cancer. We investigated the expression levels of miR-29 s in 58 GC tissues and in paracancerous tissues by qRT-PCR. The results showed that the expression of miR-29 s in GC tissues was significantly lower than that in paracancerous tissues (Fig. 3a and Supplementary Fig. 4). However, only miR-29a was closely associated with lymph node metastasis, while miR-29b and miR-29c were not significantly associated with any clinical data (Supplementary Fig. 5 and Table 1). In the 42 cases of GC with lymph node metastasis, the expression of miR-29a (C/P) was significantly lower than that in the 16 cases of GS without lymph node metastasis (P < 0.001). The expression level of miR-29a in the tumors of patients with GC demonstrated no association with age, gender, tumor size, or differentiation. We also found that high expression of miR-29a in tumors of patients with gastric cancer was associated with a longer survival time according to Kaplan–Meier survival analyses (Fig. 3b). Based on these results, we hypothesized that miR-29a may play an important role in the control of metastasis in GC.

ITGB1 plays an important role in the promotion of tumor cell metastasis. To validate this conclusion, immunohistochemistry (IHC) was used to detect the expression level of ITGB1 in the 58 carcinoma tissues (C) and noncancerous tissues (P). The results indicated that the expression level of ITGB1 in GC tissues was significantly higher than that in paracancerous tissues (Fig. 3c,d). The survival analysis revealed that patients with high expression of ITGB1 had a shorter survival time than the patients with low ITGB1 expression (Fig. 3e), which indicates that high expression of ITGB1 is closely linked to a poor prognosis of gastric cancer.

Based on the qRT-PCR results and clinical data, we found that ITGB1 was upregulated in GC, whereas the expression of miR-29a was inversely correlated (Fig. 3f). Together, these results demonstrate that high miR-29a expression with better clinical outcomes, and a lower rate of lymph node metastasis.

### miR-29a suppresses tumor cell invasion, metastasis and proliferation

**Figure 4:**
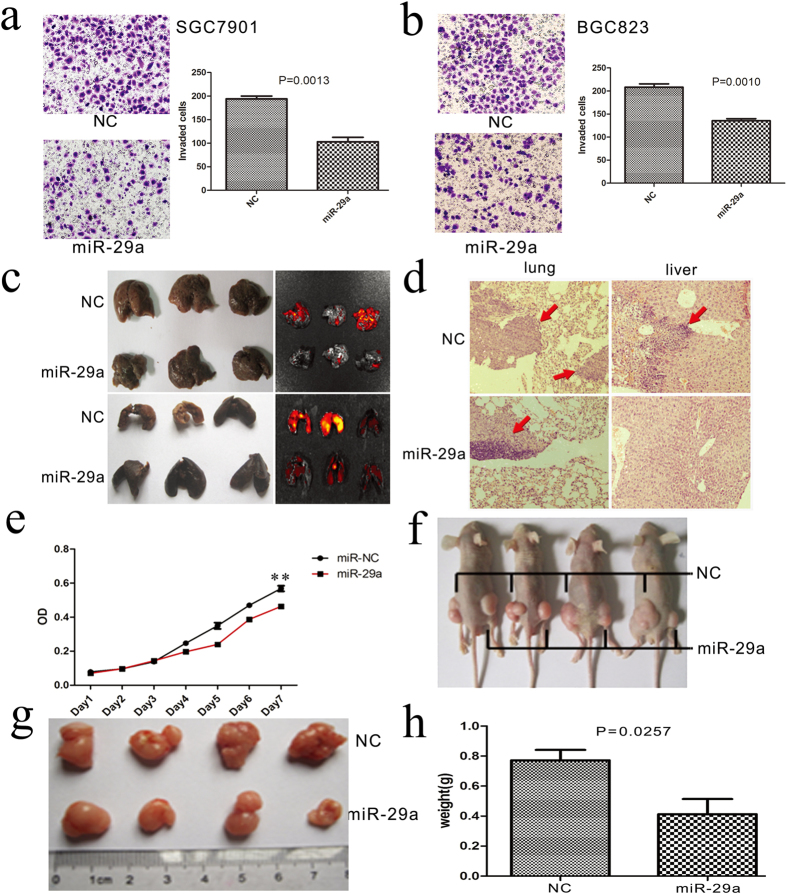
miR-29a suppresses tumor cell invasion, metastasis and proliferation. (**a,b**) A Transwell assay tested the migration ability of the GC cells when they stably express miR-29a or the control. (**c,d**) Representative fluorescence imaging and H&E staining of lungs and livers isolated from mice that received injections of SGC7901-miR-control or SGC7901-miR-29a cells. (**e**) The proliferation curve according to an MTT assay. **P < 0.01 (**f–h**) SGC7901 cell suspensions (1 × 10^6^), which were transfected with miR-29a lentivirus were injected into the right side of nude mice. The same amount of SGC7901 cells, which were transfected with negative control lentivirus, were injected into the left side of nude mice as the control. Tumors were visualized and harvested after 4 weeks. Then, tumor size (**g**) and weight (**h**) were measured.

To further study the role of miR-29a in the malignant activity of GC, SGC-7901 and BGC-823 cells were transfected with lentivirus (miR-29a and control vector, both labeled with RFP). Transwell experiments showed that overexpression of miR-29a can significantly suppress the migration of GC cells (SGC-7901 and BGC-823) (Fig. 4a,b). We next examined whether miR-29a could also suppress the metastasis of GC cells *in vivo*. We inoculated mice intravenously with SGC-7901 cells (SGC-7901-miR-29a and SGC-7901-NC) to generate lung metastasis models. The results showed that fewer and smaller metastases were detected in the lungs and livers of mice that were injected with SGC-7901- miR-29a (Fig. 4c,d). Taken together, these results showed that miR-29a functions as a suppressor of metastasis in human GC.

To investigate whether the loss of miR-29a would suppress the proliferation of GC cells, a proliferation curve generated from an MTT assay showed that miR-29 significantly inhibited the proliferation of human gastric cancer SGC-7901 cells *in vitro* (Fig. 4e). We next determined that miR-29a also suppressed the proliferation of GC cells *in vivo*. We injected SGC-7901-miR-29a and SGC-7901-NC cells subcutaneously into nude mice. The results showed that tumors in the thighs of mice that were injected with SGC-7901-miR-29a cells were smaller in size (Fig. 4f–h). Taken together, these results showed that miR-29a could suppress the malignant activity of GC cells *in vitro* and *in vivo*.

### miR-29a is required for hTERT-mediated metastasis and low expression of miR-29a promotes ITGB1 expression

As we have shown, hTERT can regulate ITGB1 expression via miR-29a. However, the mechanism of how hTERT regulates miR-29a remains unclear.

**Figure 5:**
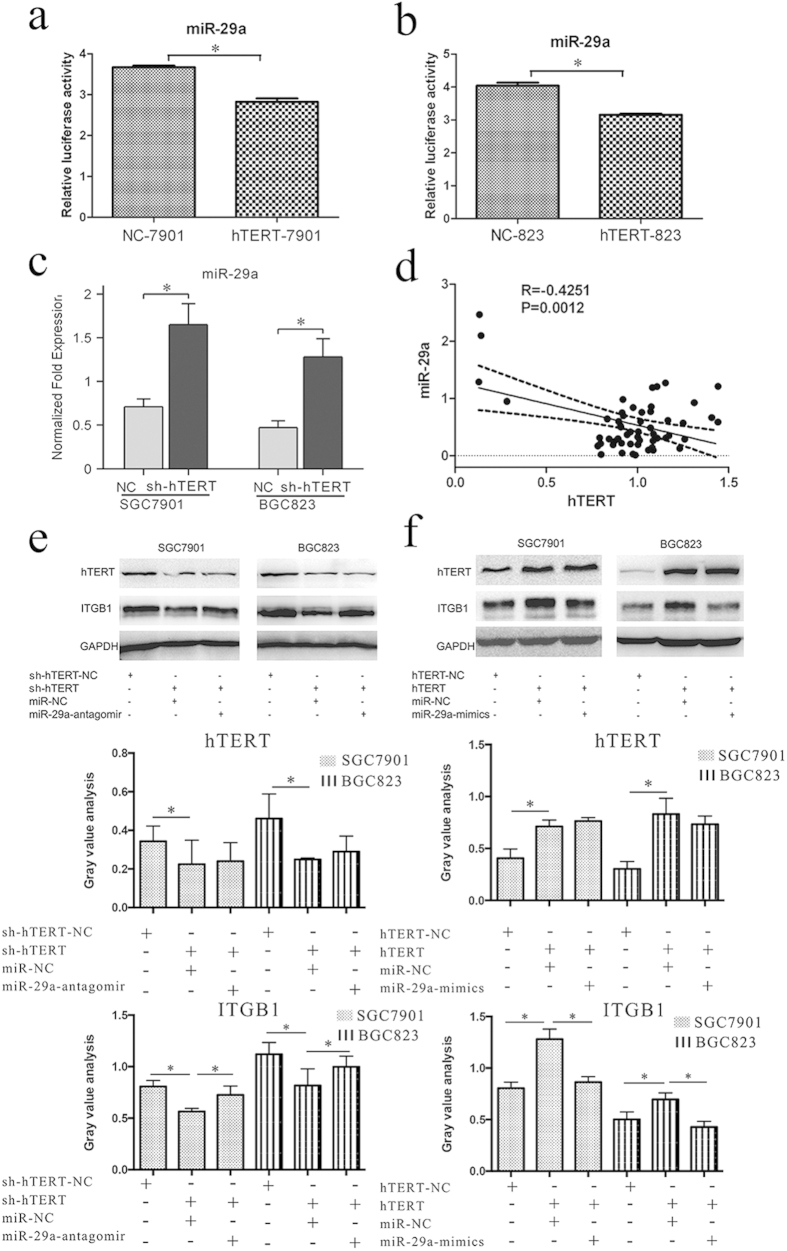
hTERT-mediated metastasis and promotion of ITGB1 expression requires miR-29a. The relative activities of the Firefly and Renilla luciferase genes were assayed in SGC-7901(**a**) and BGC-823(**b**) cells 24 h after co-transfection with TERT or control lentiviral vectors and the reporter of miR-29a (n = 3, unpaired t test). (**c**) qRT-PCR analysis of miR-29a in SGC7901 and BGC823 cells after transfection with the hTERT expression vector or the control vector. (**d**) The correlation between the levels of hTERT and miR-29a in gastric cancer and in paracancerous tissues adjacent to the cancer. (**e**) The expression level of ITGB1 after the downregulation of hTERT with or without miR-29a inhibition. Gary value analysis was used, *P < 0.05 (**f**) The expression level of ITGB1 after overexpression of hTERT with or without the restoration of miR-29a expression. Gary value analysis was used, *P < 0.05.

To check whether hTERT could regulate miR-29a, we cloned the reporter of miR-29a and co-transfected GC cells (SGC-7901 and BGC-823) with this reporter and either hTERT or control lentiviral vectors. The assays showed that hTERT significantly decreased the relative luciferase activity (Fig. 5a,b). The results of the qRT-PCR assay validated that the knock down of hTERT can increase the level of miR-29a (Fig. 5c). Statistical analysis revealed a significantly negative correlation between the expression levels of hTERT and miR-29a in GC specimens (Fig. 5d). These results demonstrated that hTERT may bind to miR-29a directly or indirectly in GC.

**Figure 6:**
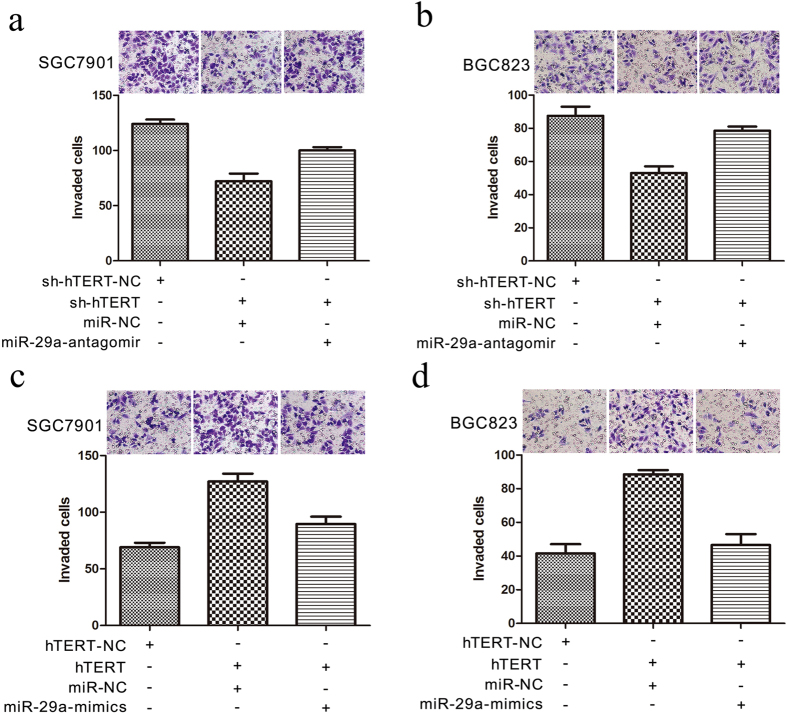
The metastatic capacity of GC cells as tested by a Transwell assay. (**a,b**) The metastatic potential of gastric cancer cells after the downregulation of hTERT with or without miR-29a knock down. (**a**) SGC7901. (**b**) BGC823. (**c,d**) The metastatic potential of gastric cancer cells after overexpression of hTERT with or without the restoration of miR-29a expression. (**c**) SGC7901. (**d**) BGC823.

To determine whether the mediation of invasive potential and the promotion of ITGB1 expression by hTERT require miR-29a, we knocked down hTERT expression in the gastric cancer cell line SGC7901 by using short hairpin construsts (Figs 5e and 6a,b). The expression level of ITGB1 and the capacity of GC cells to metastasize were decreased. Further downregulation of miR-29a in GC cells by antagomiRs abrogated the effects of hTERT downregulation; this led to a partial restoration of the ITGB1 protein level (Fig. 5e), and the invasive and metastatic potentials of gastric cancer cells were partially reversed (Fig. 6a,b). In contrast, overexpression of hTERT in gastric cancer cells promoted the expression of ITGB1 (Fig. 5f) and accelerated the invasion of gastric cancer cells. Moreover, the expression level of ITGB1 and the invasion potential of gastric cancer cells were partially decreased after the restoration of miR-29a levels by miR-29a mimics (Fig. 6c,d).

Our results indicated that miR-29a is an important downstream regulator of hTERT that controls metastasis and the expression of ITGB1 and ultimately leads to the suppression of GC metastasis. hTERT promotes the metastasis of GC cells and ITGB1 expression, at least in part, through the regulation of miR-29a.

## Discussion

In our previous study, we confirmed that hTERT is closely linked to several malignant phenotypes of cancer, such as invasion and metastasis^10^. We also found that the protein level of ITGB1 was increased after hTERT overexpression by proteomics approach^16^. Interestingly, western blot analysis confirmed that the expression of ITGB1 was significantly increased when hTERT was overexpressed in U2OS cells and in human GC cells. The expression level of hTERT was positively correlated with ITGB1 expression in clinical GC specimens, which was in accordance with the results from the proteomic analysis. Many studies have reported that ITGB1 could be regulated by microRNAs^23^,^24^, we then developed the hypothesis that whether hTERT could regulate ITGB1 via microRNAs.

Our data also demonstrated that ITGB1 is a novel target of miR-29 s. ITGB1, which is an important oncogene, plays a critical role in tumor invasion and metastasis. Many studies have indicated that ITGB1 is abnormally expressed in a variety of tumor types and that a high expression level of ITGB1 is closely associated with a poor prognosis of cancer patients^25^. Many studies have indicated that ITGB1 promotes metastasis in a variety of tumor cell types. The inhibition of ITGB1 expression might therefore reduce invasion and metastasis in lung^26^, and liver cancers^27^. The overexpression of ITGB1 increased the proliferation and metastatic potential of HepG2 cells^28^ and breast cancer cells^25^. Our data confirmed these observations as follows: the ITGB1 expression level in GC tissues was significantly higher than that in paracancerous tissues. In addition, the patients with high expression of ITGB1 had a shorter survival time than the patients with low ITGB1 expression. These results demonstrated that ITGB1 was overexpressed in tumor cells and that high expression of ITGB1 was closely linked to a poor prognosis of patients with gastric cancer. However, hTERT is not a nuclear transcription factor, which indicates that hTERT may not be able to regulate ITGB1 directly.

Recent data have indicated that miRNAs can act as important tumor promoters or suppressors. Moreover, many miRNAs have been reported to target ITGB1 and regulate ITGB1 expression. It has been reported that miR-183 can reduce ITGB1 and inhibit the metastatic potential of HeLa cells^22^. miR-124 was also reported to inhibit metastasis of oral squamous cell carcinoma^29^ and malignant glioma cells^19^ by the downregulation of ITGB1 expression. miR-134 can weaken the ability of hepatocellular carcinoma cells to metastasize both *in vitro* and *in vivo* via the direct targeting of ITGB1^30^. In breast cancer, miR-9-3p can reduce cell proliferation and invasion by the targeting of ITGB1^21^. These studies indicate that miRNAs play a critical role in the regulation of ITGB1 and in tumor metastasis.

The miR-29 family includes miR-29a, miR-29b and miR-29c^31^. The three miRNAs within the miR-29 family contain the same seed region AGCACCA. The expression of miR-29 was found to be abnormal in a variety of tumor cells and its expression is closely linked to that of a number of oncogenes, as it is involved in tumor progression^32^,^33^. MiR-29 can also target a variety of oncogenes, which results in a decrease in the proliferation of tumor cells as well as metastasis^34^,^35^.

In GC tissues, we also found that miR-29a was expressed at a significantly low level by qRT-PCR. Further analysis on the relationship between the expression level of miR-29a and the clinical data showed that low expression of miR-29a was closely associated with lymph node metastasis, whereas high expression of miR-29a in patients with gastric cancer was associated with a longer survival time. However, the correlation between tumor phenotypes and miR-29b or miR-29c was not statistically significant, despite results showed that miR-29b and miR-29c was highly expressed in GC patients (Supplementary Fig. 4 and Supplementary Fig. 5). Our results were different from those of Han *et al.*^36^, who reported that miR-29c could directly target ITGB1 expression in the regulation of gastric cancer metastasis. This difference may be due to the different clinical samples we used. Our clinical samples were from the Southwest area of China, while the samples Han and colleagues used originated in Japan and Korea. This difference may indicate that miR-29a may play a more important role in the reduction of ITGB1 expression in Chinese patients with GC. In addition, we also found that miR-29b(c) could directly target ITGB1 and that the overexpression of miR-29b(c) could decrease ITGB1 at the protein level (Supplementary Fig. 3).

Functionally, the overexpression of miR-29a could reduce the proliferative ability and the capacity for invasion of the gastric cancer cells *in vivo* and *in vitro*. This indicates that miR-29a functions as a suppressor of metastasis in human GC and is a key molecular marker in the diagnosis of early-stage gastric cancer.

Our data also revealed that ITGB1 is a novel target of miR-29 s. We therefore hypothesized that hTERT can enhance ITGB1 expression, which occurs, at least in part, by the down regulation of miR-29a expression, to promote the invasion and metastasis of gastric cancer cells. We found that miR-29a-mediated metastasis inhibition depended on the repression of its target, ITGB1. In gastric cancer cells, overexpression of miR-29a could reduce the protein expression of ITGB1. This result was further confirmed in clinical samples and in luciferase reporter assays. Thus, we demonstrated that ITGB1 is a critical downstream target of miR-29a.

As mentioned above, our miRNA microarray tests indicated that hTERT can suppress the expression of miR-29a, which was then validated in clinical samples and by qRT-PCR. More importantly, in this study, we found that the luciferase activity of miR-29a was reduced in gastric cancer cells, which were high expressed hTERT, indicating that hTERT may bind to miR-29a directly or indirectly. However, the details of this mechanism require further investigation, which will serve as a basis for our next study. In addition, we also found that hTERT requires miR-29a to mediate metastasis and promote ITGB1 expression.

In conclusion, we have demonstrated that hTERT enhances ITGB1 protein levels via the down-regulation of miR-29a expression, promoting the invasion and metastasis of gastric cancer cells. These results provide a new mechanism for our understanding of the invasion and metastasis of gastric cancer and indicate that the restoration of miR-29a may be a rational therapeutic strategy for the treatment of hTERT-mediated gastric cancer metastasis in the future.

## Materials and Methods

### Cell culture

Three human GC cell lines MKN28, BGC823 and SGC7901 cells and one human osteosarcoma cell line, U2OS, were obtained from the Type Culture Collection of the Chinese Academy of Sciences (Shanghai, China) and were cultured in standard conditions (Dulbecco’s Modified Eagle Medium (DMEM) with 10% FBS). All cells used in the experiments were in the logarithmic growth phase.

### Proteomics analysis

U2OS cells, which were infected with lenti-hTERT or lenti-Control, were harvested by using protein lysis buffer. Then, the product was concentrated and desalted in a Lab-scale TFF system (Millipore, Billerica, MA, USA). Next, the protein lysis was added into an mRP-C18 high recovery protein column (Agilent Technologies, Palo Alto, CA, USA) at 80 C with a linear multisegment gradient. Then, the product was separated using OFFGEL electrophoresis, and the peptides were analyzed by HPLC-Chip-MS/MS system consisting of a nano pump (G2226 A, Agilent). Finally, IPI human database (http://www.ebi.ac.uk/IPI) and Spectrum Mill proteomics Workbench Rev A.03.03.078 software (Agilent Technologies) was used to analysis the results.

### Animal studies

This study was performed in accordance with the NIH Animal Use Guidelines and a protocol approved by the SIU Animal Care Committee. SGC-7901 cells that were transfected with miR-29a or a miR-control lentiviral vector (labeled with RFP) were washed and resuspended in PBS (phosphate-buffered saline). In regards to the metastasis assays, 200 μl of PBS that contained 1 × 10^5^ cells was injected into the circulation through the tail vein of nude mice. The experiments were repeated in three mice. After ten weeks, the mice were sacrificed, and the livers and lungs from the mice were examined for metastases by small animal fluorescence imaging. Liver and lung tissues were also examined for metastases by hematoxylin and eosin (H&E) staining. In regards to the proliferation assays, 200 μl of PBS that contained 1 × 10^6^ cells was injected subcutaneously into nude mice. The experiments were repeated in three mice. After five weeks, the mice were sacrificed, and the tumor mass was examined for proliferation based on the number and size of the tumors.

### Clinical specimens

All experimental procedures were approved by the Institutional Review Board of the Third Military Medical University. Written informed consent was obtained for all patient samples. Fifty-eight patients who had undergone gastrectomy for gastric carcinoma at Southwest Hospital in 2003 were included in this study. None of the patients received preoperative chemotherapy. The resected specimens were histologically examined by H&E stains. The tumor tissues and corresponding non-tumor mucosal tissues were rapidly frozen in liquid nitrogen after surgical removal, and then stored at −80 °C until further use. All experiments were performed in accordance with the approved guidelines.

### Immunostaining and analysis

The immunostaining was followed by the manual introduction of VECTASTAIN^®^ Elite^®^ ABC Kit (Vector Laboratories, America). ITGB1 antibodies and hTERT antibodies were obtained from Cell Signaling Technology (CST, USA). The analysis was carried out by a image software Image-Pro Plus 6.0 (Media Cybernetics Company, America).

### Lentiviral production

Lentiviral vectors containing pre-miR-29a and an RFP reporter were purchased from GeneChem Inc (Shanghai, China). These lentiviruses were added to the GC cells using Polybrene. Stably transduced cells were then selected by FACS.

### Gene expression microarray analysis

SGC7901 lenti-hTERT and SGC7901 lenti-control were added 1 ml of Triol (Takara, Japan), respectively. The mixture were sent to CapitalBio Company for microarray analysis. Affymetrix GeneChip Human Gene 2.0 ST Array (Affymetrix, Santa Clara, CA) were used in the gene expression microarray analysis. CapitalBio Company has done the bioinformatics analysis. Microarray data was uploaded in Gene Expression Omnibus (GEO).

### Real-time qPCR

Ribobio Company (Guangzhou, China) synthesized miRNA-specific primers for miR-29a, miR-29b, miR-29c and U6. Total RNA was isolated from GC tissue and GC cells using the RNA-iso plus kit (TAKARA, Japan) and was quantified in an ultraviolet spectrophotometer (BECKMAN DU-600, USA). cDNA was synthesized using the Superscript III RT First Strand Kit (Invitrogen, USA). Real-time PCR was performed with SYBR Premix EX Taq™ II (TAKARA, Japan). The fluorescence threshold value (Ct) was calculated using the 7500 qPCR System (ABI, USA). U6 was used to normalize the miRNA expression levels.

### Western blot

Total protein was extracted by SDS (Sigma, USA). The concentration of the protein was determined by the BCA Protein Assay Kit (Beyotime, China). Western blot analyses were performed according to standard procedures. The antibodies used in this study were both purchased from Epitomics (USA).

### Luciferase assays

The 3′UTR of ITGB1 and a mutant reporter (Luc-ITGB1-mu), in which the predicted miR-29a binding site on ITGB1 was mutated, was cloned into luciferase reporters and co-transfected with either a miR-29a mimic or a control. Twenty-four h after transfection, Firefly and Renilla luciferase activities were measured by a dual-luciferase assay (Promega, USA).

### Cell Proliferation and Metastasis

An MTT Assay was performed according to the manufacturer’s instructions (Promega). The cells were plated in triplicate at the same initial density, and the OD value was recorded at 590 nm on sequential days using a plate reader (Bio-Rad). In all, 2.5 × 10^4^ cells without serum were plated in the top chamber of a 24-well Transwell plate (8-mm pore size, Corning, USA), and medium supplemented with 10% serum was used as a chemoattractant in the lower chamber. After the cells were cultured at 37 °C for 24 h, cotton wool was used to remove the non-migratory cells in the top chamber. The invading cells on the underside of the membrane were fixed in methanol for 20 min and then stained with 0.1% crystal violet for 30 min. By microscopy, three random visual fields were chosen for cell counts. The experiments were repeated in triplicate.

### Statistical analysis

Student’s t-test or the χ2 test was used to analyze miRNA and gene expression. Statistical analysis was performed with Prism 5.0 software (GraphPad). All data are presented as the means ± SE. A value of p < 0.05 was considered statistically significant.

## Additional Information

**How to cite this article**: He, B. *et al.* hTERT mediates gastric cancer metastasis partially through the indirect targeting of ITGB1 by microRNA-29a. *Sci. Rep.* **6**, 21955; doi: 10.1038/srep21955 (2016).

## Supplementary Material

Supplementary Information

## Figures and Tables

**Table 1:** The analysis of miR-29s based on the clinical data.

Clinical Pathology Data	miR-29a		miR-29b		miR-29c	
	C/P(x ± s)	P value	C/P(x ± s)	P value	C/P(x ± s)	P value
Gender						
male	0.99 ± 0.40	0.721	0.37 ± 0.047	0.115	0.40 ± 0.06	0.145
female	0.70 ± 0.14		0.93 ± 0.35		0.54 ± 0.08	
Age						
≧60	0.99 ± 0.37	0.693	0.74 ± 0.35	0.429	0.41 ± 0.08	0.456
<60	0.66 ± 0.16		0.49 ± 0.06		0.48 ± 0.06	
Lymphatic metastasis						
Yes	0.60 ± 0.07	**<0.001**	0.67 ± 0.20	0.4036	0.47 ± 0.06	0.552
No	1.4 ± 0.09		0.41 ± 0.08		0.41 ± 0.08	
Distant metastasis						
Yes	0.89 ± 0.24	0.949	1.16 ± 0.59	0.19	0.41 ± 0.05	0.148
No	0.87 ± 0.31		0.43 ± 0.05		0.58 ± 0.10	
TMN						
I/II	1.2 ± 0.65	0.965	0.39 ± 0.07	0.345	0.49 ± 0.06	0.278
III/IV	0.69 ± 0.11		0.68 ± 0.20		0.38 ± 0.06	
Size						
<5 cm	1.06 ± 0.38	0.18	0.68 ± 0.20	0.197	0.46 ± 0.05	0.814
≧5 cm	0.58 ± 0.14		0.40 ± 0.08		0.43 ± 0.09	
Differentiation						
Pooly Differentiation	0.96 ± 0.30	0.741	0.46 ± 0.06	0.141	0.44 ± 0.06	0.72
Well Differentiation	0.55 ± 0.07		0.91 ± 0.47		0.47 ± 0.07	
Survival						
Yes	2.1 ± 0.95	**0.0002**	1.06 ± 0.74	0.51	0.48 ± 0.05	0.311
No	0.51 ± 0.07		0.48 ± 0.054		0.35 ± 0.10	
