# Supplementary material for: hTERT mediates gastric cancer metastasis partially through the indirect targeting of ITGB1 by microRNA-29a
Source: Sci Rep. 2016 Feb 23;6:21955. doi: 10.1038/srep21955 (PMC4763288; doi:10.1038/srep21955)
Supplement: Supplementary Information [file srep21955-s1.pdf]

hTERT mediates gastric cancer metastasis partially through the indirect targeting of ITGB1 by microRNA-29a

Bing He [1,#], Yu-Feng Xiao [1,#], Bo Tang [1], Yu-Yun Wu [1], Chang-Jiang Hu [1], Rui Xie [1], Xin Yang [1], Song-Tao Yu [2], Hui Dong [1], Xiao-Yan Zhao [1], Ji-Liang Li [3,*], Shi-Ming Yang[1,*]

[1] Department of Gastroenterology, Xinqiao Hospital, Third Military Medical University, Chongqing, 400037, P.R. China

[2] Department of Oncology and Southwest Cancer Center, Southwest Hospital Third Military Medical University, Chongqing, 400037, P.R. China

[3] Institute of Translational and Stratified Medicine, Plymouth University Peninsula Schools of Medicine and Dentistry, The John Bull Building, 16 Research way, Plymouth PL68BU, UK.

[#] He B and Xiao YF contributed equally to this study.

[*] To whom requests for reprints should be addressed at the Department of Gastroenterology, Xinqiao Hospital, Third Military Medical University, Chongqing, 400037, P.R. China. Phone: 86-023-68755604; E-mail: ji-liang.li.@plymouth.ac.uk or shimingyang@yahoo.com.

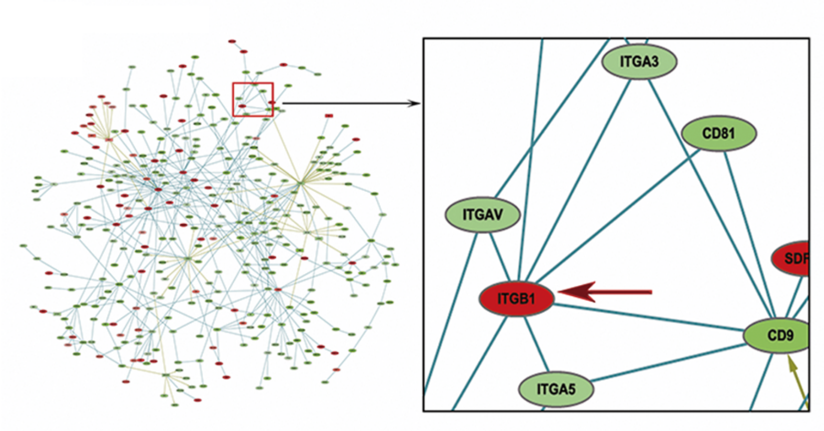

Supplementary Fig. 1 Identification of proteins related to hTERT. Proteomics techniques were used to analyze protein expression in hTERT/U2OS and EGFP/U2OS cells.

A

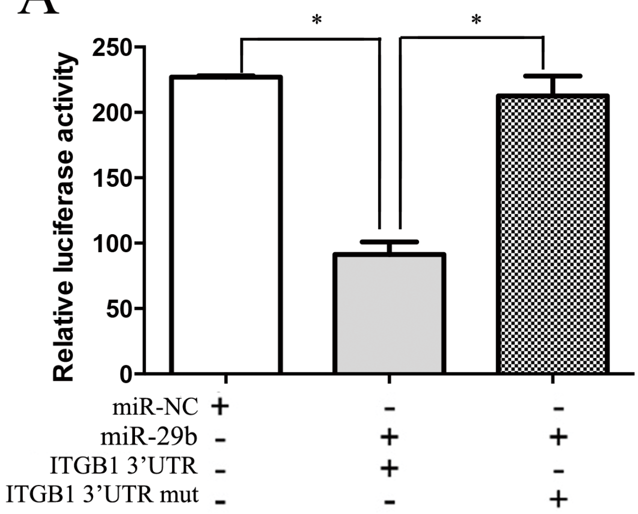

B

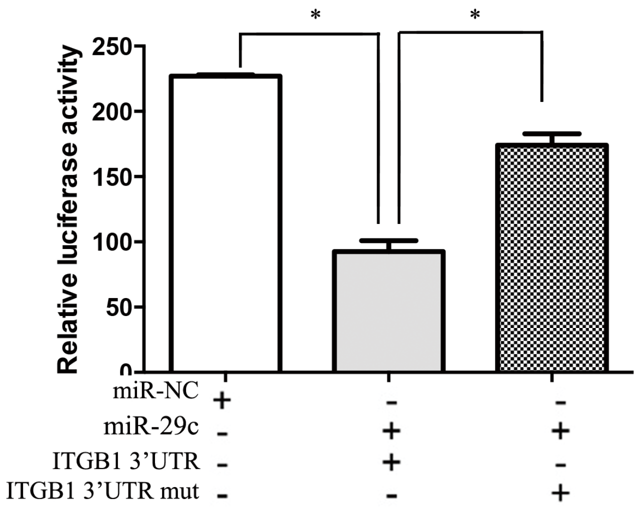

Supplementary Fig. 2 miR-29b and miR29c could also target ITGB1. The relative activities of the Firefly and Renilla luciferase genes were assayed in HEK293 cells 24 h after co-transfection with different miRNAs and the ITGB1 3'UTR or the mutated ITGB1 3'UTR (n =3, unpaired t test).

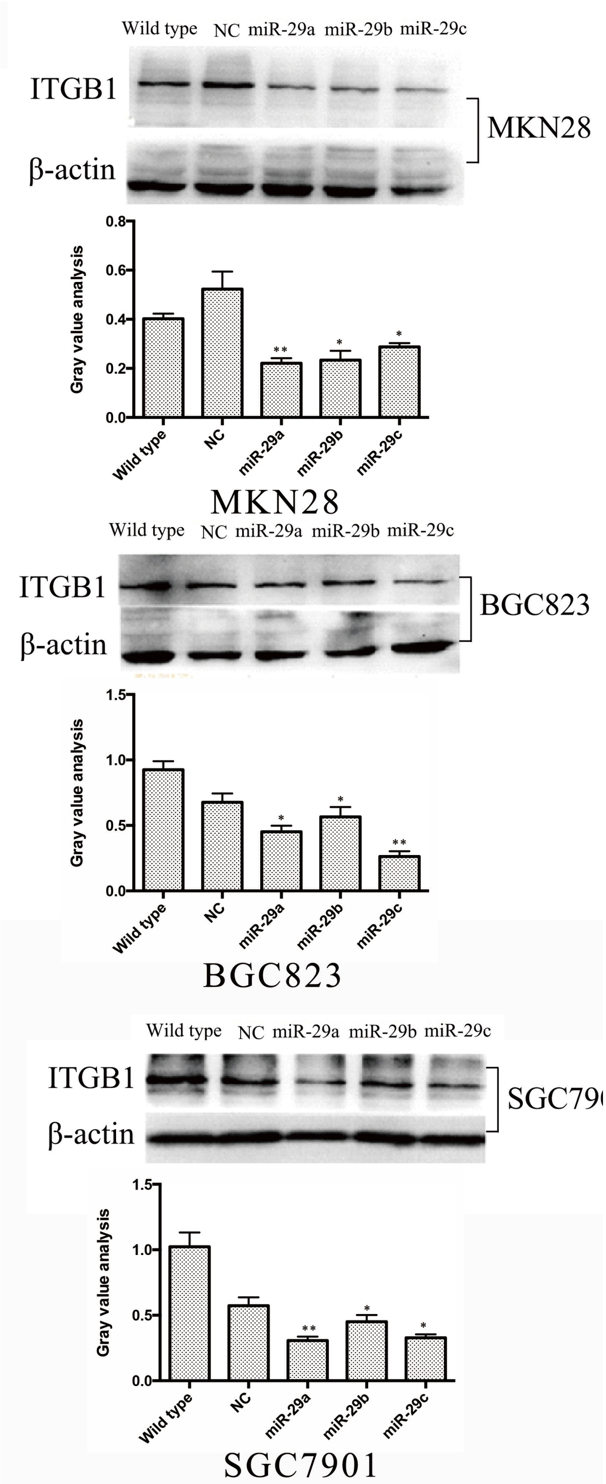

Supplementary Fig. 3 The protein level of ITGB1 in different GC cell lines was decreased when GC cells were transfected with miR-29s (miR-29a, miR-29b & miR-29c).

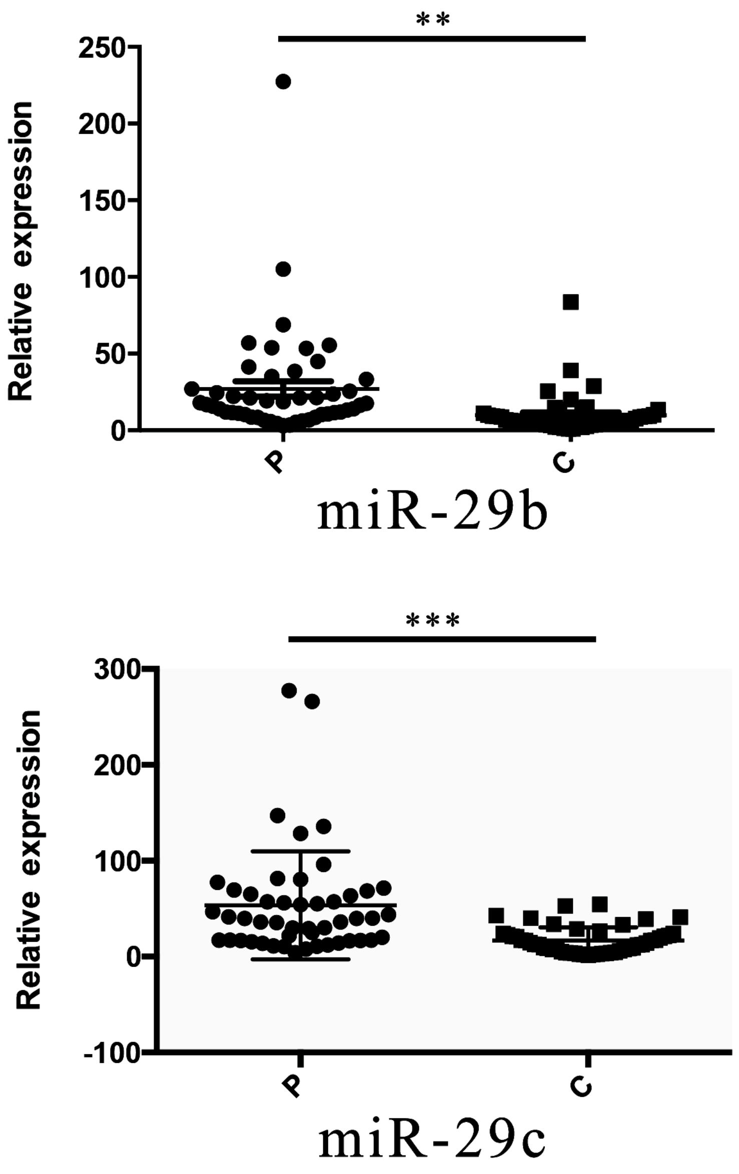

Supplementary Fig. 4 The expression levels of miR-29b and miR-29c in GC tissues and para-carcinoma tissue. ** P<0.01;*** P<0.001.

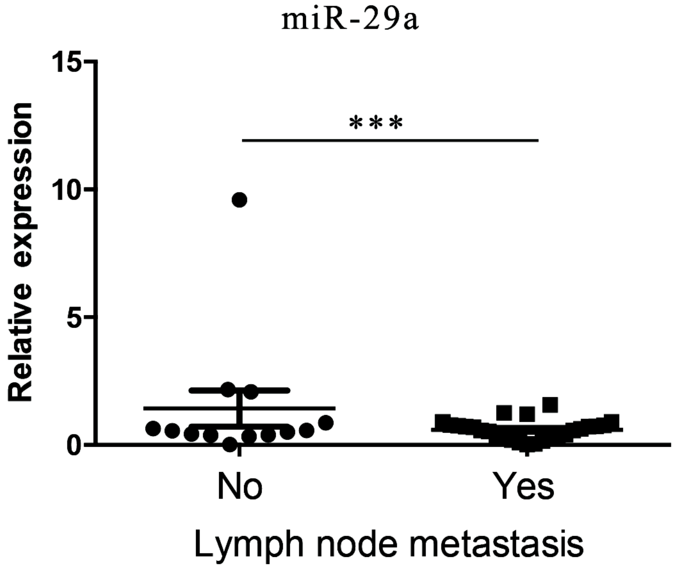

Supplementary Fig. 5 The expression levels of miR-29a in GC lymphatic metastasis tissues was compared with that of non-lymphatic metastasis tissues.***P<0.001

Supplementary Table 1 The list of 153 possible miRNAs may be regulated by hTERT in the EGFP/U2OS and hTERT/U2OS.

miRNA name	EGFP/U2OS	hTERT/U2OS
hsa-let-7a	707.7015	532.6271
hsa-let-7b	274.1716	363.9309
hsa-let-7c	344.2728	326.1634
hsa-let-7d	204.0046	229.3215
hsa-let-7e	506.7453	559.1196
hsa-let-7f	40.2544	41.84867
hsa-miR-15a	51.07143	43.76643
hsa-miR-16	648.7142	706.2369
hsa-miR-17	544.7962	616.5516
hsa-miR-18a	143.8735	137.4073
hsa-miR-19a	29.1585	23.87895
hsa-miR-19b	284.7679	215.746
hsa-miR-20a	311.2117	294.6548
hsa-miR-21	100.1862	92.12914
hsa-miR-22	341.2423	323.627
hsa-miR-23a	1012.276	914.5975
hsa-miR-24	1234.02	1199.684
hsa-miR-25	173.7522	145.66
hsa-miR-26a	467.0952	497.3513
hsa-miR-27a	376.4416	356.1029
hsa-miR-28-5p	42.62994	38.63236
hsa-miR-28-3p	29.29459	25.57476
hsa-miR-29a	191.0352	145.6852
hsa-miR-30a	39.75695	46.16138
hsa-miR-31	91.23142	113.82
hsa-miR-92a	353.59	384.2535

hsa-miR-93	446.1125	527.5379
hsa-miR-99a	73.07523	59.6944
hsa-miR-100	297.0976	297.8736
hsa-miR-103	487.3369	497.8404
hsa-miR-105	63.49211	70.5585
hsa-miR-106a	490.9032	519.1563
hsa-miR-107	405.5464	443.357
hsa-miR-197	42.41493	67.05939
hsa-miR-199a-5p	96.97963	108.2373
hsa-miR-199a-3p	65.31886	57.73526
hsa-miR-30c	151.8177	147.5773
hsa-miR-30d	73.44064	72.99141
hsa-miR-34a	180.144	207.2165
hsa-miR-181a	85.49742	116.5916
hsa-miR-181b	100.9136	112.0421
hsa-miR-199b-3p	66.37043	54.018
hsa-miR-210	56.22472	72.93665
hsa-miR-214	90.3088	152.4131
hsa-miR-221	1696. 774	1638.427
hsa-miR-222	1767.185	1911.922
hsa-let-7g	36.90584	32.18092
hsa-let-7i	94.50404	62.10943
hsa-miR-15b	170.0095	150.1459
hsa-miR-23b	437.8943	452.0891

hsa-miR-27b	64.45224	79.25587
hsa-miR-30b	76.79873	63.20047
hsa-miR-125b	267.2454	254.1004
hsa-miR-128	24.72941	29.48032
hsa-miR-130a	252.9462	252.7229
hsa-miR-138	41.22387	36.21876
hsa-miR-140-3p	118.2828	138.0498
hsa-miR-145	26.12885	26.63666
hsa-miR-152	35.76704	44.17869
hsa-miR-191	436.5224	502.485
hsa-miR-125a-5p	254.0109	319.7006
hsa-miR-126	41.62085	33.66789
hsa-miR-149	84.92864	97.83398
hsa-miR-185	125.5001	140.8873
hsa-miR-194	53.83349	47.57986
hsa-miR-320a	333.9238	464.4792
hsa-miR-106b	364.1949	353.4164
hsa-miR-301a	27.82094	33.33872
hsa-miR-99b	264.0367	340.1035
hsa-miR-296-3p	30.88685	32.32518
hsa-miR-130b	203.8818	258.6729
hsa-miR-30e	25.77642	22.91168
hsa-miR-361-5p	86.89448	104.0189

hsa-miR-362-5p	26.11207	29.88239
hsa-miR-378	42.76547	62.55178
hsa-miR-330-3p	25.77342	24.80234
hsa-miR-342-3p	34.05969	42.02005
hsa-miR-151-5p	562.2351	555.0457
hsa-miR-151-3p	123.3334	123.3334
hsa-miR-331-3p	34.50319	31.58218
hsa-miR-324-5p	56.16293	67.09329
hsa-miR-324-3p	30.22456	50.24999
hsa-miR-339-5p	129.2243	162.34
hsa-miR-339-3p	44.96498	47.55272
hsa-miR-345	31.63338	27.776
hsa-miR-423-5p	38.98057	43.94865
hsa-miR-423-3p	184.791	225.9024
hsa-miR-425	130.9673	163.7231
hsa-miR-20b	52.62603	41.72286
hsa-miR-483-5p	26.74907	25.91998
hsa-miR-484	32.25549	32.74816
hsa-miR-494	108.2787	122.9456
hsa-miR-193b	161.6832	202.2108
hsa-miR-181d	35.16217	40.32467
hsa-miR-500	31.0488	29.20397
hsa-miR-502-3p	28.75347	38.92744

hsa-miR-532-5p	27.53349	34.26889
hsa-miR-532-3p	30.64588	31.09514
hsa-miR-455-3p	142.7856	174.7371
hsa-miR-92b	69.42234	89.42196
hsa-miR-574-3p	55.47237	70.45915
hsa-miR-625	51.48642	63.02607
hsa-miR-627	27.17437	28.28687
hsa-miR-629	36.79043	36.14176
hsa-miR-638	246.2426	235.9132
hsa-miR-663	58.27912	58.20403
hsa-miR-421	22.77095	28.45872
hsa-miR-671-5p	31.04451	31.06446
hsa-miR-767-5p	52.16331	51.27911
hsa-miR-1224-5p	32.51545	34.88644
hsa-miR-320b	369.855	471.4009
hsa-miR-320c	331.7775	424.8726
hsa-miR-1271	28.0655	27.08658
hsa-miR-1301	28.64591	35.06239
hsa-miR-768-5p	100.1884	67.97903
hsa-miR-768-3p	62.40017	62.40017
hsa-miR-708	69.39626	73.33089
hsa-miR-744	92.56998	103.454
hsa-miR-923	1585.663	1491.78

hsa-miR-935	24.81308	29.39483
hsa-miR-1180	26.97575	36.15508
hsa-miR-1234	27.96088	24.24099
hsa-miR-1207-5p	102.6847	115.559
hsa-miR-1302	22.72545	24.74886
hsa-miR-1246	66.93472	60.09637
hsa-miR-1249	44.39766	31.71293
hsa-miR-1253	24.33099	24.33099
hsa-miR-1258	23.77175	21.46041
hsa-miR-1268	78.85004	78.00222
hsa-miR-1275	43.48922	53.64387
hsa-miR-1280	39.41599	25.73146
hsa-miR-1308	5448.917	1451.78
hsa-miR-1307	58.76749	61.30853
hsa-miR-720	70.34725	38.50874
hsa-miR-320d	194.1261	210.4553
hsa-miR-1826	3212.972	2672.433
hsa-miR-17-star	26.99915	26.47205
hsa-miR-21-star	36.09521	46.20903
hsa-miR-25-star	27.86162	28.63975
hsa-miR-27a-star	24.34952	30.90107
hsa-miR-93-star	37.79513	41.37652
hsa-miR-181c-star	30.83521	32.80639

hsa-miR-132-star	35.14151	35.42126
hsa-miR-138-1-star	26.46217	33.32797
hsa-miR-149-star	166.4829	155.0294
hsa-miR-106b-star	67.14909	73.55842
hsa-miR-99b-star	32.36349	35.42126
hsa-miR-500-star	29.00356	27.69816
hsa-miR-92b-star	29.79002	31.42701
hsa-miR-589-star	34.3749	26.67057
hsa-miR-550-star	31.08964	34.03331
hsa-miR-625-star	30.21133	22.32658
hsa-miR-1228-star	92.62067	76.28333
